# Long-Term Creep Behavior of the Intervertebral Disk: Comparison between Bioreactor Data and Numerical Results

**DOI:** 10.3389/fbioe.2014.00056

**Published:** 2014-11-20

**Authors:** A. P. G. Castro, C. P. L. Paul, S. E. L. Detiger, T. H. Smit, B. J. van Royen, J. C. Pimenta Claro, M. G. Mullender, J. L. Alves

**Affiliations:** ^1^Center for Mechanical and Materials Technologies, Department of Mechanical Engineering, University of Minho, Guimarães, Portugal; ^2^INSIGNEO Institute for in silico Medicine, Department of Mechanical Engineering, University of Sheffield, Sheffield, UK; ^3^Department of Orthopaedic Surgery, VU Medical Center, Amsterdam, Netherlands; ^4^Research Institute MOVE, Faculty of Human Movement Sciences, VU Medical Center, Amsterdam, Netherlands; ^5^Skeletal Tissue Engineering Group Amsterdam, VU Medical Center, Amsterdam, Netherlands; ^6^Department of Plastic, Reconstructive and Hand Surgery, VU Medical Center, Amsterdam, Netherlands

**Keywords:** intervertebral disk, loaded disk culture system, custom finite element solver, creep behavior, circadian variations

## Abstract

The loaded disk culture system is an intervertebral disk (IVD)-oriented bioreactor developed by the VU Medical Center (VUmc, Amsterdam, The Netherlands), which has the capacity of maintaining up to 12 IVDs in culture, for approximately 3 weeks after extraction. Using this system, eight goat IVDs were provided with the essential nutrients and submitted to compression tests without losing their biomechanical and physiological properties, for 22 days. Based on previous reports (Paul et al., 2012, 2013; Detiger et al., 2013), four of these IVDs were kept in physiological condition (control) and the other four were previously injected with chondroitinase ABC (CABC), in order to promote degenerative disk disease (DDD). The loading profile intercalated 16 h of activity loading with 8 h of loading recovery to express the standard circadian variations. The displacement behavior of these eight IVDs along the first 2 days of the experiment was numerically reproduced, using an IVD osmo-poro-hyper-viscoelastic and fiber-reinforced finite element (FE) model. The simulations were run on a custom FE solver (Castro et al., 2014). The analysis of the experimental results allowed concluding that the effect of the CABC injection was only significant in two of the four IVDs. The four control IVDs showed no signs of degeneration, as expected. In what concerns to the numerical simulations, the IVD FE model was able to reproduce the generic behavior of the two groups of goat IVDs (control and injected). However, some discrepancies were still noticed on the comparison between the injected IVDs and the numerical simulations, namely on the recovery periods. This may be justified by the complexity of the pathways for DDD, associated with the multiplicity of physiological responses to each direct or indirect stimulus. Nevertheless, one could conclude that ligaments, muscles, and IVD covering membranes could be added to the FE model, in order to improve its accuracy and properly describe the recovery periods.

## Introduction

The intervertebral disk (IVD) is a highly inhomogeneous porous structure, which contains solid and fluid materials. It is, in its majority, avascular. The central structures of the disk, the nucleus pulposus (NP), and the annulus fibrosus (AF), are quite different in both constitution and function, but are paired structures, vertically limited by the cartilage (CEPs) and vertebral (VEPs) endplates (Raj, 2008; Shankar et al., 2009). The NP presents a gel-like structure with embedded fibers and occupies the core of the IVD. Surrounding it, emerge an amount of concentrically arranged fibers supported on a porous matrix, which is the AF (Urban, 2000; Shankar et al., 2009; Hollingsworth and Wagner, 2011). The CEP is a layer of hyaline cartilage that is responsible for most of the nutrients exchange with the adjacent vertebral body (VB). One IVD and two VB constitute the motion segment (MS), which is the functional unit of the spine (Ebraheim et al., 2004; Raj, 2008; Schmidt and Reitmaier, 2013).

The correct identification of the IVD components and its healthiness has benefited from the advances on the medical imaging field. The IVDs are positioned in one of the most sensitive locations of the human body, next to the spinal canals and in close proximity to its nerve roots. Consequently, *in vivo* studies are quite challenging to perform (Nachemson and Morris, 1964). As a matter of fact, the work of Wilke et al. (1999) proved that those studies could be performed on humans, but they are highly dependent on the availability of volunteers, particularly because these experiments can be excessively painful and intrusive. Therefore, the major part of the information about the IVD behavior still comes from *in vitro* studies, using human or animal IVDs. These studies are usually performed *ex vivo*, but some up-to-date techniques allow the IVD to be kept alive after the sacrifice of the animal. The works of Chan et al. (2013), Gantenbein et al. (2006), Haglund et al. (2011), Korecki et al. (2007), Paul et al. (2012, 2013), and Walter et al. (2014) described bioreactors capable of providing nutrition and mechanical stimulation to the extracted IVD between 1 and 3 weeks. These versatile bioreactors are becoming the next benchmark for IVD experimental studies. Allied to these remarkable developments, numerical studies through finite element analysis (FEA) are benefiting from the improvements on the computational power to become more exhaustive and wide-ranging every day (Schmidt et al., 2013; Castro et al., 2014).

**Figure 1:**
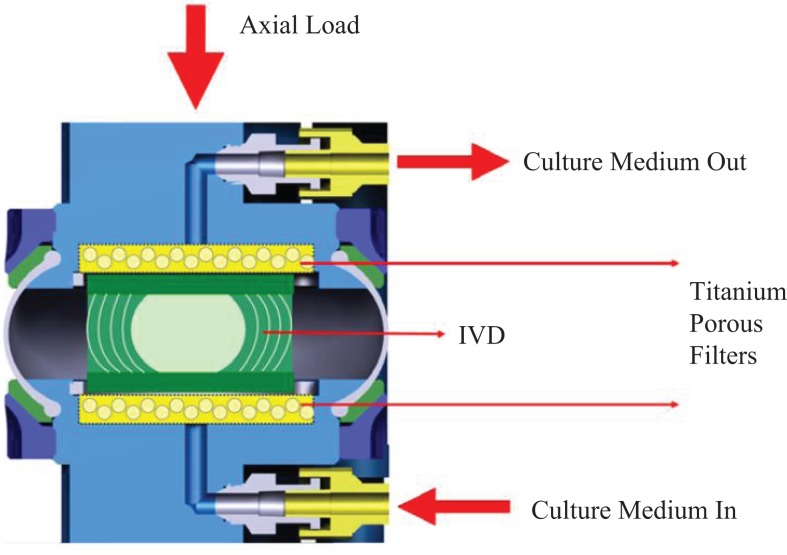
**Schematic representation of the LDCS**. Adapted from Paul et al. (2012).

This work primarily deals with the analysis of the bioreactor data from the Department of Orthopedic Surgery of VUmc (Amsterdam, The Netherlands). The bioreactor system was developed within this group, and is denominated as loaded disk culture system (LDCS). In short, this system is capable of maintaining an IVD alive for at least 3 weeks once extracted, after the sacrifice of the animal, which is usually a goat. In comparison with the abovementioned analogous bioreactors, this is the one, which reports the longest period of IVD viability. The loading feature allows the IVD to be submitted to compression tests without losing its biomechanical and physiological properties (Paul et al., 2012, 2013). A schematic representation of this system is shown in Figure 1.

The LDCS reproduces the IVD native environment, by enabling the close monitoring of oxygen and nutrient supply levels, through the introduction of a culture medium, along with providing static or dynamic mechanical axial loading, as seen in Figure 1. The culture medium is pumped through a semi-porous titanium filter underneath the IVD at a speed of 3 ml/h and an osmolarity of 360–380 mOsm (Paul et al., 2012). The previous LDCS-related publications reported that, from the physiological point of view, a dynamic loading regime cause large dynamic displacement, while the static regimes induced a prolonged creep effect (Paul et al., 2012, 2013). Up to 12 IVDs may be simultaneously under experiment in this equipment.

This is a feasibility study, whose key goal is to numerically reproduce the LDCS experiments through FEA, using the custom tools developed on the Center for Mechanical and Materials Technologies of the University of Minho (CT2M-UM, Guimaraes, Portugal) (Alves et al., 2010; Castro et al., 2013, 2014; Cavalcanti et al., 2013).

## Materials and Methods

The object of study is an LDCS dataset, which contained the results of experiments performed with eight partial lumbar MS, i.e., one IVD, the two adjacent VEPs, and the connective membranes. In detail, four goats provided one control (“Cont”) and one injected (“Inj”) IVD each, i.e., one IVD was maintained intact during the lifetime of the animal (non-injected) and the adjacent IVD was injected with 0.5 U/ml 100 μL of chondroitinase ABC (CABC) on PBS solution. The CABC is an enzyme that cleaves proteoglycans, i.e., this PBS-CABC compound is injected on the goat IVD with the aim of triggering degeneration (Detiger et al., 2013; Adams et al., 2014; Stefanakis et al., 2014). The lumbar IVDs used in the current study are derived from goats, which were also used in the study published by Detiger et al. (2013). Although the individual IVDs were not used for analyses in that study, the entire lumbar spinal segments of the goats were scanned with MRI pre-operatively and 12 months after CABC injection. This protocol intended to confirm degeneration of the IVDs due to CABC, using the Pfirmann scale (Pfirrmann et al., 2001; Griffith et al., 2007). Detiger and co-workers reported a large inter-individual variation for all parameters measured. However, no significant difference in Pfirmann scale was measured for the IVDs included in the current study (data not previously published).

The partial MS were tested under physiological loading conditions, which consisted of a sinusoidal load (1 Hz) of 150 N average and 100 N amplitude for 16 h (activity period), followed by other sinusoidal load (1 Hz) of 50 N average and 10 N amplitude for 8 h (resting period). Sinusoidal loadings are associated with dynamic loading regimes (Qasim et al., 2012). This loading profile is comparative to activities such as lying down and walking in goats and relaxed standing and non-supported sitting in humans. It must be highlighted that the transition between the activity and resting periods is performed with 1 h of triangular loading (0.25 Hz) of 200 N average and 100 N amplitude, which is also a dynamic loading regime (Hoogendoorn et al., 2008; Paul et al., 2012, 2013; Detiger et al., 2013).

**Figure 2:**
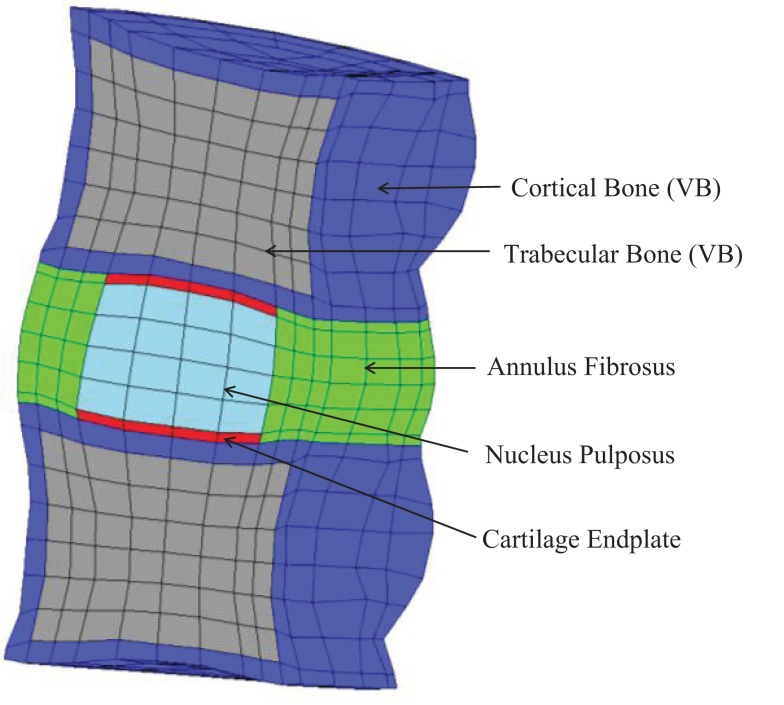
**Sagittal cut of the partial human L3–L4 MS FE model, which contains 1892 27-node quadratic hexahedral elements and 16,425 nodes**.

The comparative finite element (FE) simulations were performed with the partial MS FE model shown in Figure 2, which included L3 and L4 VB (without facets) and the L3–L4 IVD. Ligaments and muscles were not considered, as they were also removed from the partial MS tested on the LDCS. The most relevant material properties of this model are summarized in Table 1, accordingly to the state-of-the-art of soft tissue and IVD constitutive modeling (Dreischarf et al., 2014; Freutel et al., 2014). For a more detailed description of the custom FE solver, the biphasic IVD constitutive modeling and MS FE model, the authors would like to refer to Castro et al. (2013, 2014). It must be highlighted that AF fibers’ mechanical properties are assumed to evolve linearly through the axial plane, both in radial and circumferential directions (Eberlein et al., 2001), as previously described by Cavalcanti et al. (2013). Fiber angle also varies from ±23.2° at ventral position to ±46.6° at dorsal position, in accordance with Holzapfel et al. (2005).

**Table 1:** **Material properties of the partial physiological MS FE model**.

		NP	AF	CEP	TB	CB
Isotropy, Mooney Rivlin model (Schmidt et al., 2007)	*C*_10_ (MPa)	0.15	0.18	1.00	41.67	3846.15
	*C*_01_ (MPa)	0.03	0.045	0.00	0.00	0.00
Permeability, van der Voet model (Argoubi and Shirazi-Adl, 1996; Ferguson et al., 2004)	$K_{0}^{*}\left(mm^{4}N^{-1}s^{-1}\right)$	7.5e−4	7.5e−4	7.5e−3	1.0e−1	1.0e−1
	*M*	8.50	8.50	8.50	18.0	22.0
Anisotropy, Holzapfel model (Holzapfel et al., 2005)	$\bar{k}$	–	300.0	–
	*k*_4_ = *k*_6_ (MPa)	–	12.0	–
Viscoelasticity, Generalized Maxwell model (Iatridis et al., 1997; Ehlers et al., 2009)	*a*_1_	1.7	–
	τ_1_ (s)	11.765	–
	*a*_2_	1.2	–
	τ_2_ (s)	1.100	–
	*a*_3_	2.0	–
	τ_3_ (s)	0.132	–
Biphasic Swelling, Wilson model (Galbusera et al., 2011)	*R* (N mm mmol^−1^ K^−1^)	8.31450	8.31450	–
	*T* (K)	298.0	298.0	–
	ϕ_int_	0.83	0.83	–
	ϕ_ext_	0.92	0.92	–
	*C*_ext_ (mmol mm^−3^)	0.00015	0.00015	–
	*C*_F,0_ (mmol mm^−3^)	0.00030	0.00018	–
	*n_f_*_,0_	0.80	0.70	–

*Multiple data sources were assessed, as stated on each entry of the table. Isotropic (MS ground substances), permeability, anisotropic (AF fibers), viscoelastic, and swelling properties were considered*.

In order to compare the human IVD FE model with the LDCS data, some important assumptions had to be held, since the LDCS experiments were performed with goat IVDs (Schmidt and Reitmaier, 2013; Reutlinger et al., 2014). The average axial cross section of the goat IVD is around 300–400 mm^2^ (Paul et al., 2012), accordingly to the experimental data, while the average axial cross section of the human IVD stands between 1200 and 1600 mm^2^. The IVD of the current FE model has an axial cross section of 1555.3 mm^2^ (Castro et al., 2014).

Consequently, the axial cross section of the human IVD is averagely four times larger than the goat IVD, so one could assume that the loads to be applied on the MS FE model should be four times greater than the loads experimentally applied on the goat IVDs, on the LDCS. In what concerns to the IVD height, the FE model has an average height of 12 mm, while the goat IVDs have an average height of 9 mm, which means that no normalization is needed. Previous studies verified that human and goat IVDs produce similar internal stresses, regardless of the geometric differences (Ayotte et al., 2001; Alini et al., 2008; Hoogendoorn et al., 2008; Schmidt and Reitmaier, 2013).

The other major simplification is related with the loading regimes. At the hourly time-scale, it was numerically verified that the sinusoidal loadings applied in the LDCS and the equivalent static average loadings produce comparable numerical outcome loadings. Since the sinusoidal wave meant additional computational effort, one adopted the lighter static and constant loadings.

Two daily cycles were simulated, after a preconditioning period of 8 h, for osmotic equilibration before loading. The numerical physiological loading profile consists of 600 N for 16 h (activity period), followed by 200 N for 8 h (resting period). This model is known as “Native” (Galbusera et al., 2011; Paul et al., 2012). The transition between the activity and resting periods is performed with 800 N for 1 h, in order to maintain a similar timeline with the experimental tests.

The effect of the PBS-CABC compound was numerically modeled through the reduction of the osmotic pressure gradient. As aforementioned, CABC cleaves proteoglycans, which are involved in the osmotic pressure mechanisms, through the regulation of the fixed charged density (*C*_F_). In detail, proteoglycans have an essential role on fluid attraction and detainment, which allows the IVD to maintain its internal pressure, namely due to the “vessel-wall”-like behavior of the AF fibers. Hence, if the proteoglycan content is reduced, the hydration of the IVD is also reduced and the osmotic pressure gradient is lower (Wognum et al., 2006; Massey et al., 2012; Stefanakis et al., 2014). The initial fixed charge density (*C_F_*_,0_) of the NP was then reduced, in two levels, until it reached the initial fixed charge density of the AF, which remain unaltered (Table 1). This dual-step reduction is presented on Table 2. The model with the intermediate *C_F_*_,0_ reduction is entitled “Low OsmP 1” and the model with the *C_F_*_,0_ value equal to the AF’s properties is denominated “Low OsmP 2.” Finally, in order to evaluate an extreme situation of proteoglycans cleavage, i.e., total split, a model without osmotic swelling was also tested (“No Swelling”).

**Table 2:** **Osmotic swelling material properties and correspondent initial osmotic pressure of both native and reduced OsmP FE models**.

Model	NP *C_F_*_,0_ (mmol mm^−3^)	Initial NP OsmP (MPa)
Native	0.00030	0.189
Low OsmP 1	0.00024	0.106
Low OsmP 2	0.00018	0.036
No Swelling	–	0.000

The other core hypothesis to simulate degeneration was the modification of the tissues’ permeability, but this mechanism is highly dependent on the degeneration grade. Mild degeneration, as reported by Detiger et al. (2013), stands for grades III–IV on Thompson’s scale (Thompson et al., 1990; Sivan et al., 2013). Low to mild degeneration are probably related to a decrease of CEP permeability, due to the calcification of this component, while mild to severe degeneration are more likely associated with an increase of CEP permeability, as a result of probable crack openings (Urban, 2000; Adams and Dolan, 2012; Stefanakis et al., 2014). The permeability variations on the degenerated NP and AF remain uncertain, but some studies pointed out that permeability could increase (Iatridis et al., 1998; Urban, 2000; Johannessen and Elliott, 2005; O’Connell et al., 2011). The proteoglycans might also be related to the permeability variations, but such association is also unclear. The IVD components are also described to increase in stiffness, as modeled by Natarajan et al. (2006) and Schmidt et al. (2007), but stiffness variations were not previously described in the literature as having a direct link to the injection of PBS-CABC compound (Detiger et al., 2013).

## Results

**Figure 3:**
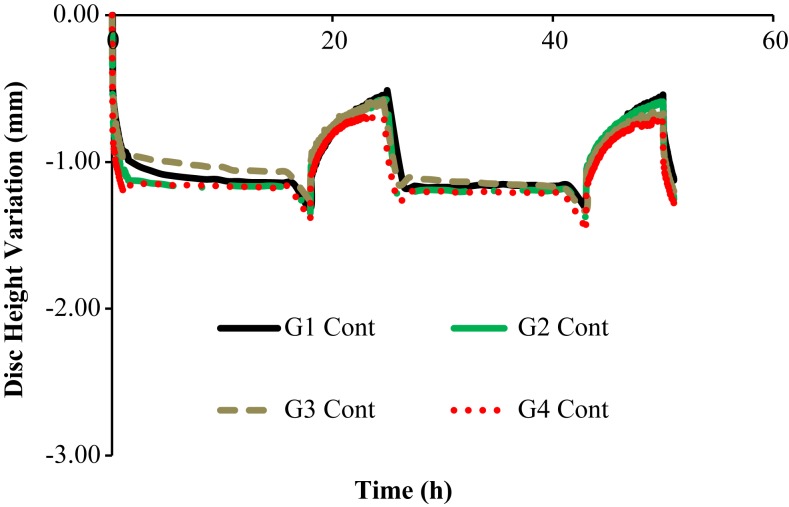
**Disk height variation outcomes of the LDCS, for two daily cycles, regarding the control goat IVDs**.

**Figure 4:**
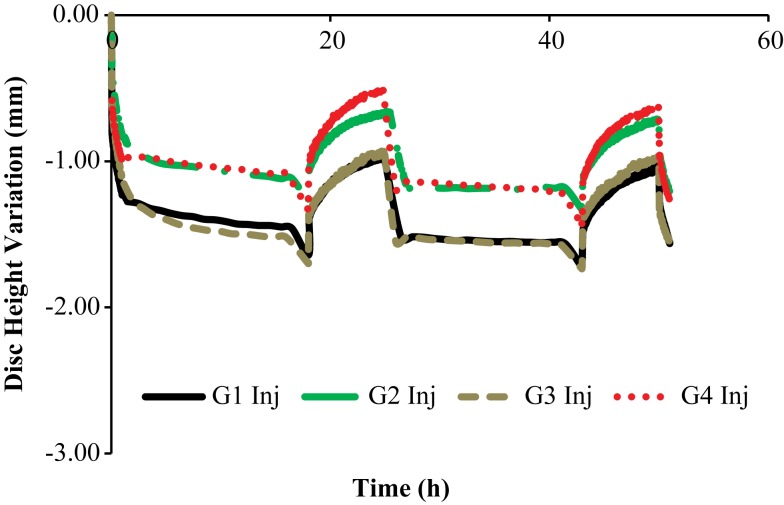
**Disk height variation outcomes of the LDCS, for two daily cycles, regarding the injected goat IVDs**.

**Figure 5:**
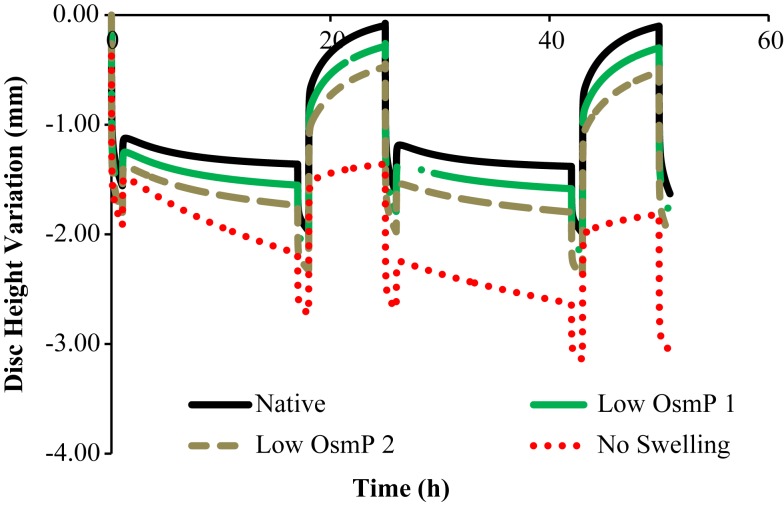
**Disk height variation outcomes of the four numerical approaches to the experimental data, namely “Native,” “Low OsmP 1,” “Low OsmP 2,” and “No Swelling” models**.

**Figure 6:**
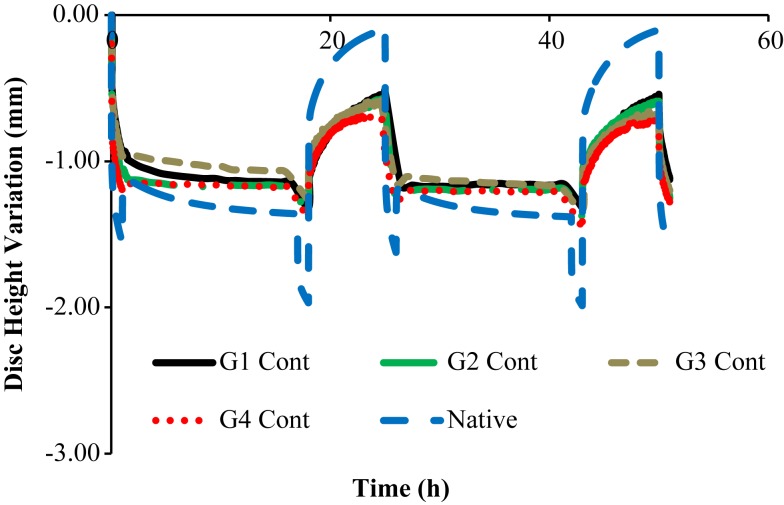
**Native DHV outcomes of the experimental and numerical tests, for two daily cycles, i.e., the four control goat IVDs are here compared with the native numerical model**.

**Figure 7:**
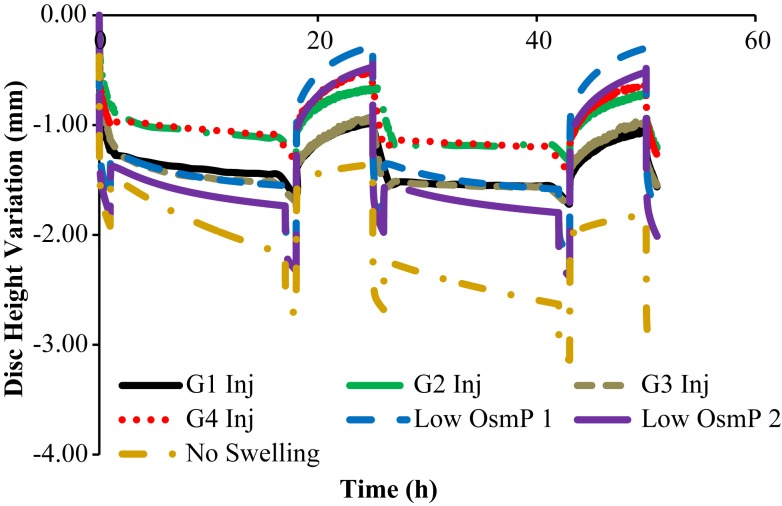
**Non-physiological DHV outcomes of the experimental and numerical tests, for two daily cycles, i.e., the four injected goat IVDs are here compared with the two reduced OsmP and the “No Swelling” models**.

Figures 3 and 4 show the disk height variation (DHV) of the control and injected goat IVDs on the LDCS, respectively. The timeline was limited to two daily cycles. Other IVD-related outputs could be extracted from the FE solver, namely intradiscal pressure, fiber elongation of volume variation. Figure 5 shows the DHV along the test’s timeline for the five numerical approaches, namely “Native,” “Low OsmP 1,” “Low OsmP 2,” and “No Swelling” models. Finally, Figure 6 shows the comparison between the physiological DHV outcomes of the experimental and numerical tests (“Control” and “Native,” respectively). Figure 7 shows the comparison between the injected IVDs DHV results of the experimental and the corresponding numerical tests, i.e., the two reduced OsmP models and the “No Swelling” model. Table 3 summarizes the DHV values of these seven DHV curves, demonstrating the differences between the four experimental tests, and also between the experimental tests and the numerical ones.

**Table 3:** **Summary of the DHV values of the four injected IVDs and the reduced OsmP models, after each activity and recovery period, obtained by numerical simulation**.

IVD		DHV (mm)			
		First activity period	First recovery period	Second activity period	Second recovery period
Experimental	G1 inj	−1.46	−0.96	−1.57	−1.05
	G2 inj	−1.12	−0.68	−1.18	−0.72
	G3 inj	−1.52	−0.92	−1.56	−0.97
	G4 inj	−1.09	−0.49	−1.21	−0.63
Numerical	Low OsmP 1	−1.56	−0.29	−1.59	−0.30
	Low OsmP 2	−1.74	−0.47	−1.80	−0.49
	No Swelling	−2.17	−1.36	−2.63	−1.82

## Discussion

The control IVDs tend to maintain their height along the experiment, while the injected IVDs tend to lose height progressively (Figures 3 and 4, respectively). This systematic height reduction is mostly noticed on the loss of the ability to return to the same height level after the recovery period. Such findings are in agreement with the work of Detiger et al. (2013), in which consistent evidence of IVD height reduction after the injection of the CABC compound was found. However, some discrepancies were noticed between the injected IVDs, which can be justified by the inter-specimen variability of the goats. In fact, goats 1 and 3 seem to be more sensitive to the CABC compound than goats 2 and 4.

In what concerns to Figure 5, it is noticeable that the behavior of the numerical model corresponds to what was theoretically expected. It must be highlighted that the transition periods are excluded from the current analysis of the maximum and minimum displacement measurements, as their purpose was just to establish the transition between the activity and recovery periods, on the experimental apparatus. Firstly, the “Native” model is able to fully recover the initial height on the resting periods, and the maximum DHV is inside the physiological displacement range. Secondly, the reduced OsmP models show some signs of degeneration, as the fluid flow seems to be diminished, and thus the initial height is not recovered. This effect is progressive, as the “Low OsmP 1” model is less affected than the “Low OsmP 2” model. The maximum displacement also progressively increased (from the first to the second daily cycle), in comparison with the “Native” model. In addition, the “No Swelling” model shows clear signs of degeneration, as the maximum displacement is significantly increased and this increase is progressive throughout the daily cycles, without any sign of recovery. The recovery rate shall be understood as the difference between the DHV after the activity period and the DHV after the recovery period. The lowest recovery rate was registered by the “No Swelling” model, i.e., approximately 0.80 mm for the two daily cycles, meaning that this is the model with less capacity for regaining fluid content. At the light of the experimental tests, the behavior of the “No Swelling” model probably means that the proteoglycan cleavage promoted by the injected quantity of PBS-CABC compound is only enough to cause mild degeneration, as expected. Considering the described outcomes, the situation of an IVD without any osmotic pressure gradient is certainly related to severe degeneration (Adams et al., 2014; Neidlinger-Wilke et al., 2014).

The comparison of the first two daily cycles of the native IVDs with the equivalent period of the “Native” model (Figure 6) shows that the numerical model is able to reproduce the physiological behavior of the goat IVD, particularly during the activity period. Numerically, the maximum DHV was −1.36 mm, while the average experimental measurement was −1.20 mm, considering the four native goat IVDs.

In what concerns to the resting periods, an important difference is noticed, as the numerical model is able to regain all the fluid lost during the activity period. The goat IVDs do not reach that DHV value of approximately 0.00 mm, as their average DHV value on the resting period is −0.58 mm.

Nonetheless, these four control IVDs maintain the same DHV recovery level from the first to the second daily cycle, which was previously described as a sign of no degeneration. In other words, the DHV results indicate no degeneration, but incomplete recovery. This fact is probably related with the intrinsic behavioral differences between the goat and human IVDs, namely the specific biomechanical stimuli. The nutrition pathways included in the LDCS functioning and the ion-influenced fluid retention may also have a role in this situation. The numerical model helped to understand that the ideal situation is to fully recover the fluid on the resting periods. However, the action of AF fibers may also be limiting the range of DHV, and this limit situation (maximum extension of the fibers) is not predicted in the FE solver. This action shall not be considerably different in human and goat spines.

Figure 7 shows that the “Low OsmP 1” model is closer to the injected IVDs from goats 1 and 3 than the “Low OsmP 2” model. The native IVDs presented a DHV difference of averagely 0.58 mm after the recovery periods, when compared with the “Native” model. The average difference between “G1 Inj” and “G3 Inj” and the “Low OsmP 1” model, after the recovery periods, is 0.50 mm, i.e., the distance between these two injected IVDs and “Low OsmP 1” is close to what was measured for the native condition, which denotes consistency of the outcomes. In addition, the analogous difference for the maximum displacement is averagely 0.25 mm, which is in agreement with the 0.16 mm difference found for the native condition. The DHV measurements from the injected IVDs of goats 2 and 4 may not be directly compared with these numerical outcomes, as their maximum displacement is significantly lower than the analogous measurement of the other two injected IVDs. The DHV measured after each recovery period is nearer to the numerical models, but the recovery rate is considerably uneven. Nevertheless, the average recovery rate of “G4 Inj” is inside the 0.50–0.60 mm range of the other two injected IVDs. The behavior of the “No Swelling” model is significantly distant from these injected IVDs.

On the numerical point of view, the results suggest that the MS FE model is excessively sensitive to the applied loads, having that the goat IVDs present a much more limited range of height variation. If these experimental tests were performed *in vivo*, one might question the amount of influence of the surrounding tissue (ligaments) on IVD mechanical properties and measurements. However, these structures were removed from the IVDs before the LDCS tests. On the one hand, one shall not neglect the influence of the external membranes covering each MS, since they also play a role on limiting the range of motion of IVD and other organs (Humphrey, 2003). On the other hand, previous studies from Ayotte et al. (2001) and Van der Veen et al. (2005) suggested that the fluid flow through VEP and CEP was modified once the IVD was extracted from the animal and/or tested isolated, which could cause the distorted recovery periods. Therefore, the behavioral differences are probably related to the intrinsic biochemical and biomechanical properties of the goat IVDs, including the action of the removed MS components.

On the experimental point of view, the injection of the PBS-CABC compound on the goats caused degeneration on the targeted IVDs, but it could affect the adjacent levels similarly. The comparison with the MS FE model was successful, particularly for the physiological situations (control). The degenerated behavior of the IVD is extremely complex, given to the undisclosed physiological criteria related to the diverse possible loading and environmental stimuli. The degeneration prevention mechanisms are still to be fully understood (Adams and Roughley, 2006; Haschtmann et al., 2006; Qasim et al., 2012; Detiger et al., 2013; Galbusera et al., 2013; Adams et al., 2014; Benneker et al., 2014).

To sum up, this work substantiated that the LDCS is capable of mimicking the IVD in-body environment and also that the FE model is able to reproduce the experimental outcomes (control and injected). The successful association between these two distinct approaches to the IVD study allowed a very much detailed description of the proposed physiological and non-physiological situations.

In terms of future work, it would be interesting to simulate the full duration of the LDCS tests, i.e., up to 3 weeks of creep simulation with the IVD FE model. More computational resources would be needed to fulfill this task. In addition, ligaments, muscles, and IVD covering membranes would be an effective addition to the FE model, as their role is relevant for the global behavior of each MS.

## Conflict of Interest Statement

The authors declare that the research was conducted in the absence of any commercial or financial relationships that could be construed as a potential conflict of interest.
